# Early Clinical Manifestations and Visual Prognosis of Japanese Patients with Norrie Disease: A Case Series and Literature Review

**DOI:** 10.3390/jcm15187324

**Published:** 2026-09-21

**Authors:** Takuma Futami, Yu Higashi, Itsuka Matsushita, Tomoko Tsukahara-Kawamura, Satoshi Ueki, Takaaki Hayashi, Masatoshi Hirayama, Sachiko Nishina, Noriyuki Azuma, Shunji Kusaka, Hiroyuki Kondo

**Affiliations:** 1 Department of Ophthalmology, University of Occupational and Environmental Health, Kitakyushu 807-8555, Japan; futataku_uoeh@yahoo.co.jp (T.F.); youhigasi@gmail.com (Y.H.); i-matsushita@med.uoeh-u.ac.jp (I.M.); 2 Department of Ophthalmology, Fukuoka University, Fukuoka 814-0180, Japan; ttsukahara@adm.fukuoka-u.ac.jp (T.T.-K.); mhirayama@fukuoka-u.ac.jp (M.H.); 3 Division of Ophthalmology and Visual Science, Graduate School of Medical and Dental Sciences, Niigata University, Niigata 950-2181, Japan; sueki@med.niigata-u.ac.jp; 4 Department of Ophthalmology, The Jikei University School of Medicine, Tokyo 105-8461, Japan; takaaki@amy.hi-ho.ne.jp; 5 Division of Ophthalmology, National Center for Child Health and Development, Tokyo 157-8535, Japan; nishina-s@ncchd.go.jp (S.N.); azuma-nkt@yacht.ocn.ne.jp (N.A.); 6 Department of Developmental and Regenerative Biology, Institute of Science Tokyo, Tokyo 152-8550, Japan; 7 Department of Ophthalmology, Faculty of Medicine, Kindai University, Osaka 577-8502, Japan; skusaka@gmail.com

**Keywords:** Norrie disease, familial exudative vitreoretinopathy, retinal detachment, vitrectomy, NDP

## Abstract

**Background/Objectives:** To characterize the early clinical manifestations and treatment outcomes of Japanese patients with Norrie disease. Retrospective multicenter observational case series with a systematic literature review. **Methods:** Fourteen infants with genetically confirmed Norrie disease were retrospectively reviewed. Clinical findings, disease stage, interocular asymmetry, treatment, and visual outcomes were evaluated using a modified staging system based on familial exudative vitreoretinopathy (FEVR) and the International Classification of Retinopathy of Prematurity. A systematic review of previously reported Japanese cases was performed to assess disease severity at initial diagnosis. **Results:** Twenty-eight eyes of 14 patients were included. The median age at the initial examination was 2.5 months. Twenty-three eyes (82%) had stage 5 retinal detachment, whereas only five eyes (18%) had stage 2 or 4 disease. Interocular asymmetry was uncommon. Twenty-one eyes (75%) underwent laser photocoagulation and/or surgery. Visual outcomes were poor; at the most recent follow-up, all but two stage 4 eyes treated with laser photocoagulation had no light perception. Combined analysis of our cohort and the literature review identified 29 Japanese patients from 19 families and demonstrated that most already had advanced bilateral retinal detachment within the first two months of life. **Conclusions:** Norrie disease typically presents with advanced bilateral retinal detachment during early infancy, leaving limited opportunities for effective intervention. Early recognition and genetic diagnosis are therefore essential.

## 1. Introduction

Norrie disease is a rare X-linked recessive disorder characterized by congenital retinal detachment, hearing loss, and intellectual disability. It was first described by Norrie in 1927 [1]. The clinical features of 48 affected individuals from nine families were systematically characterized by Warburg in 1961 [2], who established the eponymous designation of Norrie disease. Norrie disease is caused by pathogenic variants in the Norrie disease protein (*NDP*) gene, which encodes the secreted protein norrin. The causative gene was identified by Chen et al. [3]. Norrin binds to the WNT receptor complex and activates the canonical β-catenin signaling pathway, known as the norrin/β-catenin signaling pathway. In addition to Norrie disease, *NDP* variants are also known to cause other retinal disorders, including X-linked familial exudative vitreoretinopathy (XL-FEVR).

Norrie disease is typically detected because of leukocoria or poor visual fixation [4]. Ocular manifestations include total retinal detachment caused by tractional retinal detachment and retinal dysplasia presenting as pseudoglioma. As retinal detachment progresses, patients may lose light perception and eventually develop end-stage ocular changes, such as corneal opacity and obliteration of the anterior chamber. Clear ophthalmic diagnostic criteria distinguishing Norrie disease from XL-FEVR have not yet been established [4,5]. Although the visual prognosis is generally extremely poor, one report described preservation of visual function to at least light perception following vitrectomy performed very early after birth [6]. However, few studies have evaluated the clinical findings in a large number of patients specifically from the perspective of how early treatment must be initiated to achieve a favorable outcome.

In Japan, Norrie disease was first reported by Fujita et al. in 1979, and only a limited number of additional cases have subsequently been described [7,8,9,10,11,12,13]. We have previously reported several Japanese patients with Norrie disease, focusing on individual case presentations and *NDP* genotypes [14,15,16]. Although the present study includes some previously reported patients, it analyzes them from a different perspective by focusing on age at presentation, disease severity at presentation, and treatment outcomes. In addition, we systematically reviewed all previously reported Japanese cases to provide a comprehensive overview of the early clinical manifestations of Norrie disease in Japan. To characterize disease severity at presentation, we evaluated patients using a novel clinical staging system based on existing classification schemes for advanced FEVR and retinopathy of prematurity (ROP).

## 2. Materials and Methods

### 2.1. Classification of Norrie Disease Patients

This study was conducted in accordance with the tenets of the Declaration of Helsinki. The study protocol was reviewed and approved by the institutional review boards of the University of Occupational and Environmental Health Hospital (Project code SE10-01, 23-080), Kindai University (28-264, 22-132, R05-177), the Jikei University School of Medicine (24-231 6997), the National Center for Child Health and Development (518), and Fukuoka University (U21-04-015). Written informed consent was obtained from all participants or their legal guardians. The study included patients with Norrie disease in whom pathogenic *NDP* variants had been identified through genetic testing performed at the University of Occupational and Environmental Health Hospital or Fukuoka University Hospital. The cohort comprised 12 patients from 10 families whose genotypes had been previously reported [14,16], three patients from two families whose cases are currently under review for publication, and one previously unreported patient. Genotypes were determined by Sanger sequencing, as previously described [14].

Ophthalmic evaluations included assessments of visual acuity, slit-lamp biomicroscopy, fundus examination, ocular ultrasonography, fluorescein angiography, and ultrasound biomicroscopy.

**Figure 1:**
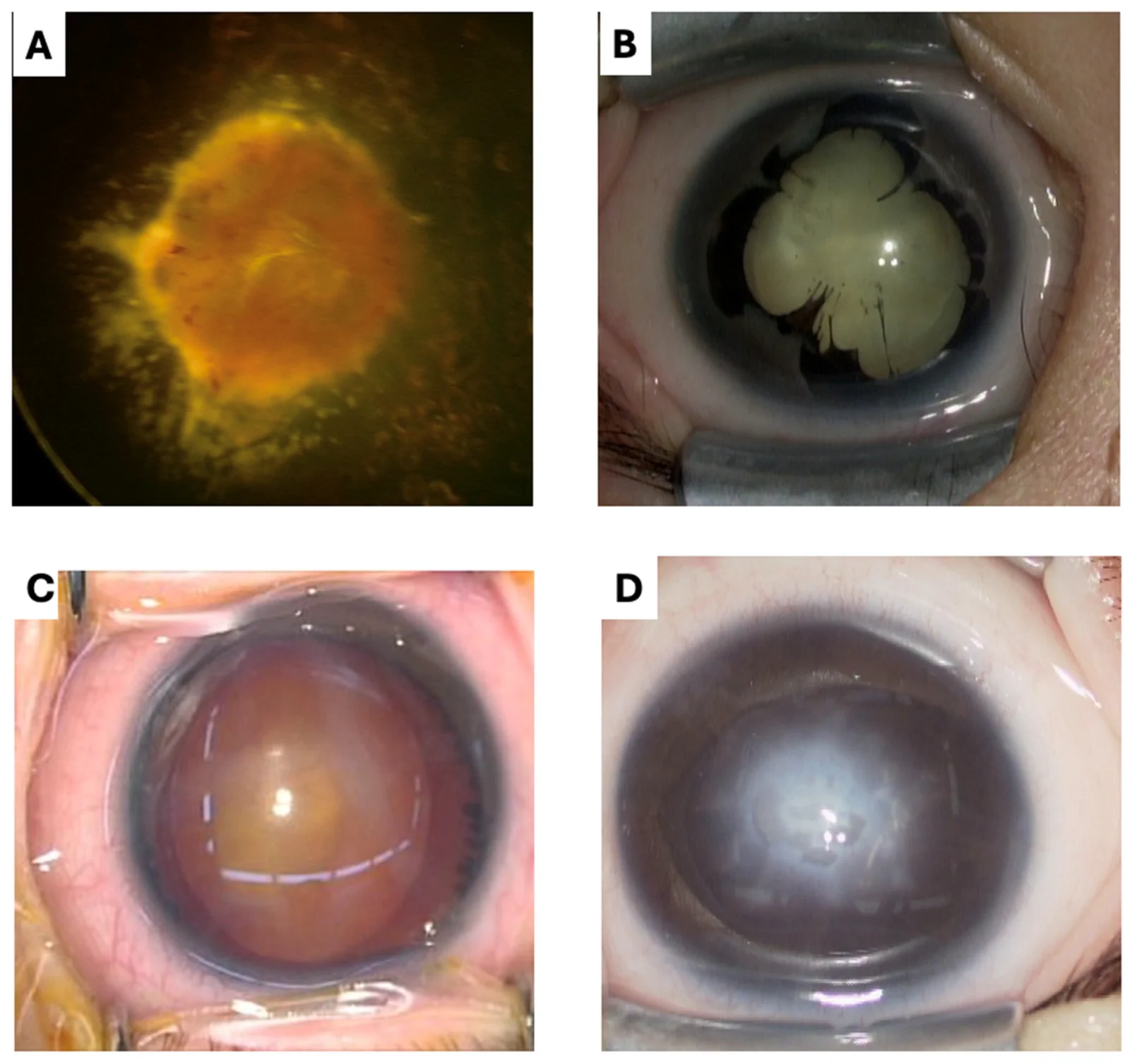
**Representative clinical findings in Norrie disease.** (**A**) Fundus photograph of the right eye of Patient 4-2 showing retinal dysplasia, the characteristic “pumpkin” lesion of Norrie disease associated with partial retinal detachment (stage 4). Retinal vessels are poorly visualized around the dysplastic retina. (**B**–**D**) External photographs of advanced Norrie disease. (**B**) Left eye of Patient 1 showing posterior synechiae with a preserved anterior chamber (stage 5B). (**C**) Right eye of Patient 3 showing marked mydriasis associated with neovascular glaucoma (stage 5C). (**D**) Right eye of Patient 1 showing corneal opacity with obliteration of the anterior chamber (stage 5C). Panel A corresponds to stage 4. Stage 5 is subclassified based on reference [18].

Disease severity was classified using a simplified version of the FEVR staging system [17], in which the original A/B subclassification was omitted. Stage 1 was defined as peripheral avascular retina; stage 2, peripheral avascular retina with retinal neovascularization; stage 3, partial retinal detachment sparing the macula; stage 4, partial retinal detachment involving the macula or congenital absence of macular formation without total retinal detachment; and stage 5, total retinal detachment (Table 1). The original FEVR stage 5A/5B subclassification (open- and closed-funnel total retinal detachment) was replaced with the International Classification of Retinopathy of Prematurity, Third Edition (ICROP3) classification [18]. Accordingly, stage 5 was subdivided into three categories: stage 5A, total retinal detachment with the optic disc visible on ophthalmoscopy; stage 5B, total retinal detachment with the optic disc obscured by retrolental fibrovascular tissue or a closed-funnel retinal detachment; and stage 5C, end-stage disease characterized by secondary glaucoma associated with anterior lens displacement and/or corneal opacity (Table 1). The presence or absence of retinal dysplasia, the characteristic “pumpkin” lesion of Norrie disease (Figure 1) [6], and persistent fetal vasculature (PFV) were also evaluated. Disease stage, interocular asymmetry, and clinical manifestations were analyzed according to age at the initial ophthalmic examination.

### 2.2. Survey Methods

**Figure 2:**
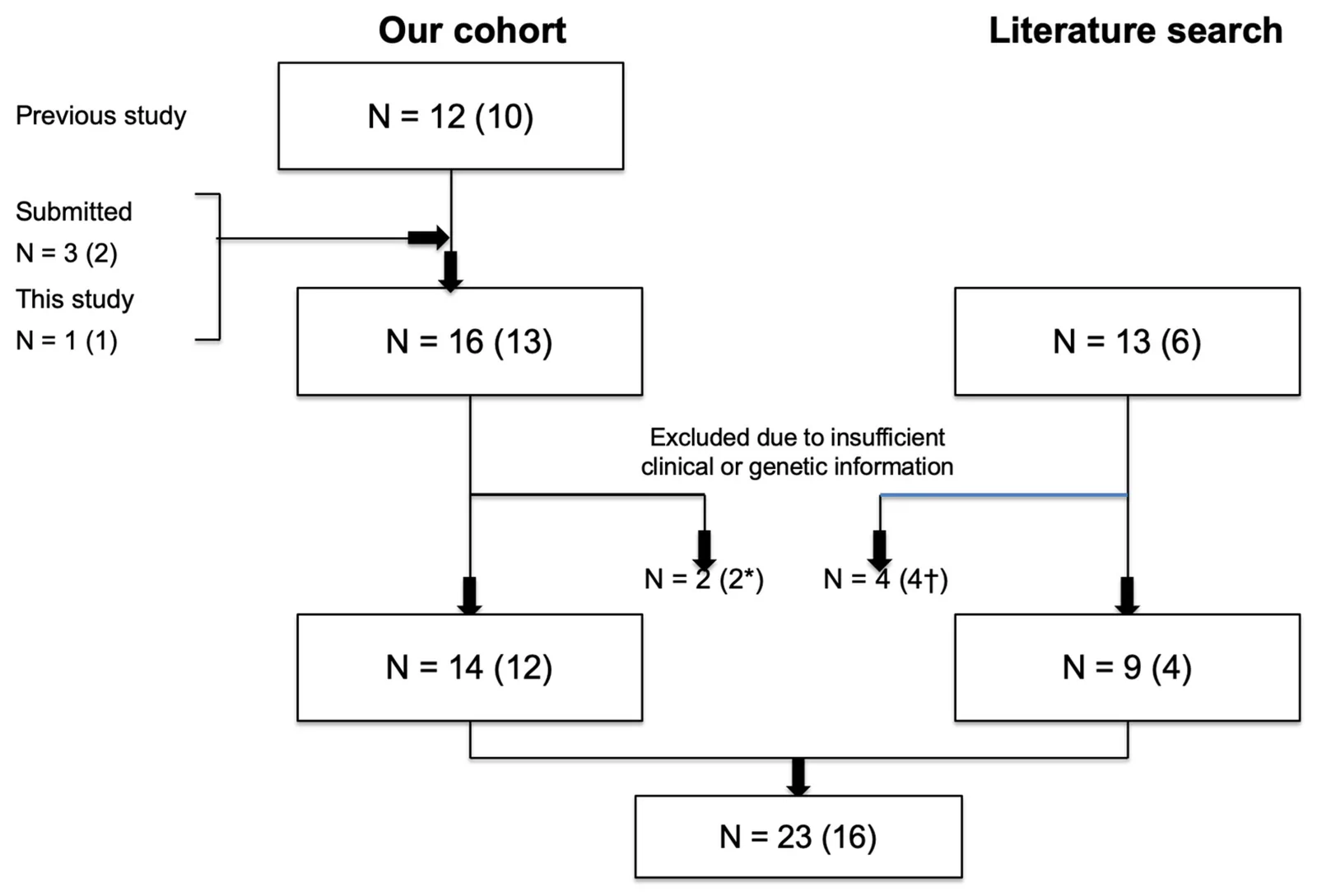
**Flowchart of patients and families included in the study.** N = number of patients; the number in parentheses indicates the number of families. * Another member of the same family was included in this study. † One patient without genetic testing and three patients from three families with insufficient clinical information were excluded; other members of two of these families were included in this study.

Patients were excluded from the analysis of acute-stage clinical manifestations if the age at the initial examination or detailed ophthalmic findings were unavailable or if the first ophthalmic examination was performed during adulthood (Figure 2).

To identify all reported Japanese patients with Norrie disease, a systematic literature review was performed using PubMed (http://www.ncbi.nlm.nih.gov/pubmed/, accessed 15 January 2026) and Ichushi-Web (https://search.jamas.or.jp/, accessed 15 January 2026). Both databases were searched in January 2026 using the term “Norrie disease.” Reported cases with genetically confirmed *NDP* variants were included, whereas patients without genetic confirmation were excluded. Clinical data, including age at presentation and disease stage, were extracted for analysis.

**Novel case.** A 1-month-old boy, born at term with a normal birth weight, was referred for ophthalmic evaluation because of left upper eyelid swelling. Slit-lamp examination revealed shallow anterior chambers in both eyes and an iris bombe in the right eye. The corneal diameters were 9 mm in the right eye and 10 mm in the left eye. Fundus examination was not possible because of opaque ocular media. B-scan ultrasonography demonstrated highly reflective vitreous lesions suggestive of vitreous hemorrhage in both eyes and a cord-like lesion in the left eye. The patient was initially diagnosed with bilateral PFV. However, fluorescein angiography of the patient’s mother revealed peripheral retinal vascular abnormalities suggestive of FEVR. Subsequent genetic testing identified an initiation codon variant in the *NDP* gene (NM_000266.4), c.3G>A (p.Met1?), establishing the diagnosis of Norrie disease. The patient also had a family history of hemophilia A, which was also diagnosed at 0 months old. He currently has no light perception in either eye.

## 3. Results

### 3.1. Acute-Phase Clinical Findings

Acute-phase clinical findings were available for 28 eyes of 14 patients (Table 2 and Figure 2). The age at the initial ophthalmic examination ranged from birth to 3 years, with a median age of 2.5 months. Among the 28 eyes, corneal opacity was observed in seven eyes of six patients (25%), and corneal edema was observed in one eye of one patient (4%). The anterior chamber was obliterated in five eyes of four patients (18%), shallow in 15 eyes of 10 patients (54%), and of normal depth in eight eyes of five patients (29%). Regarding iris and pupillary findings, marked mydriasis associated with neovascular glaucoma was observed in five eyes of four patients (18%), whereas miosis with posterior synechiae was observed in five eyes of three patients (18%). Based on the FEVR staging system, with stage 5 subclassified according to the International Classification of Retinopathy of Prematurity, Third Edition, no eyes were classified as stage 1, stage 3, or stage 5A. Stage 2, stage 4, stage 5B, and stage 5C disease were observed in one eye of one patient (4%), four eyes of four patients (14%), 15 eyes of 12 patients (54%), and eight eyes of seven patients (29%), respectively. Among the eight eyes with stage 5C disease and corneal opacity, two retained a deep anterior chamber (Figure 1). In all eight eyes with stage 5C disease (29%), fundus visualization was not possible because of leukocoria.

**Figure 3:**
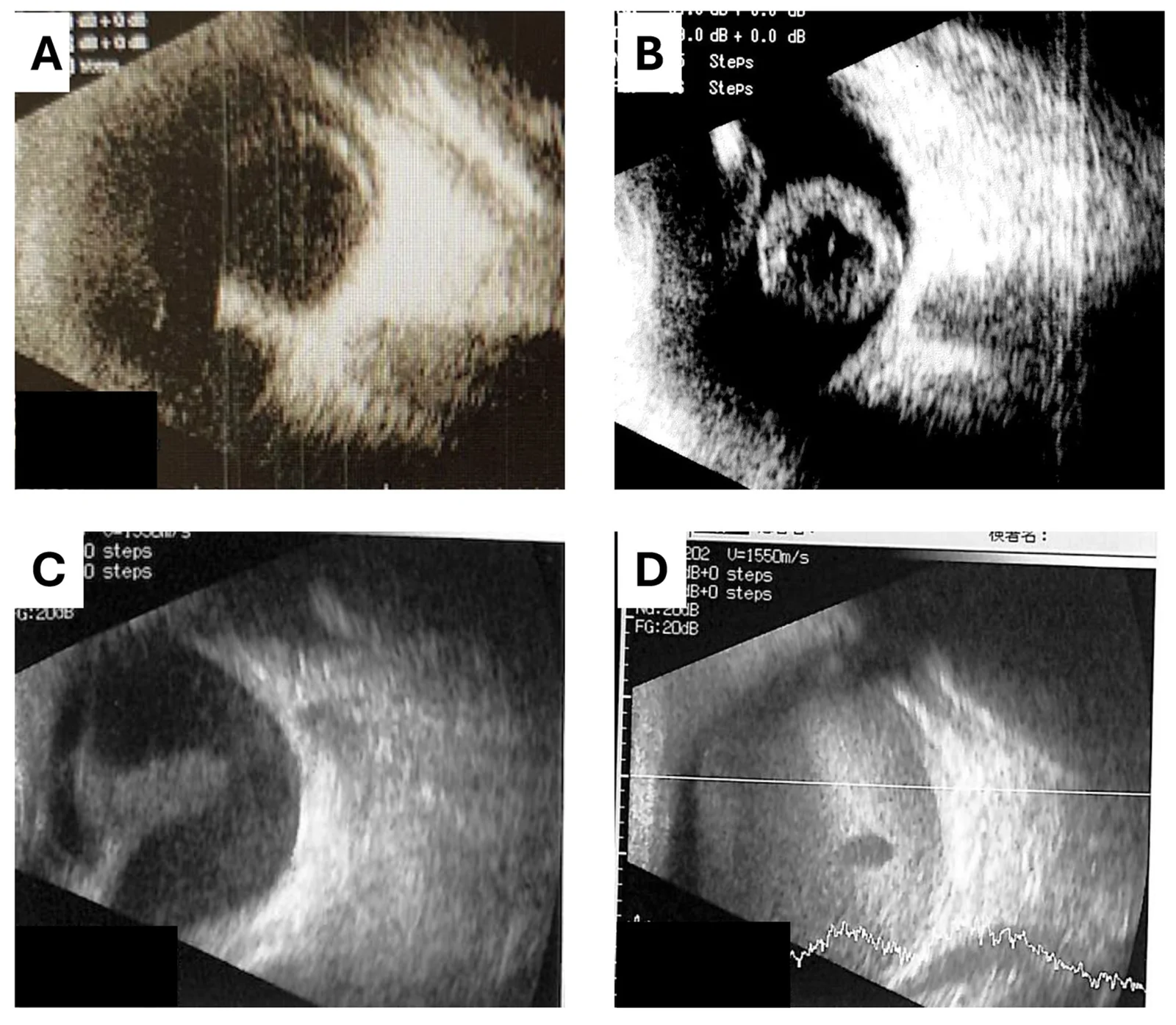
**Representative B-scan ultrasonography findings in Norrie disease.** (**A**–**D**) B-scan ultrasonography images from the right eye of Patient 11 (**A**), the right eye of Patient 5 (**B**), and the left (**C**) and right (**D**) eyes of Patient 3. (**A**) Partial retinal detachment associated with a PFV-like stalk. (**B**,**C**) Open- and closed-funnel retinal detachments, respectively. (**D**) Diffuse high echogenicity caused by advanced vitreoretinal degeneration and subretinal hemorrhage, precluding accurate assessment of the retinal configuration.

B-scan ultrasonography was performed in 23 eyes of 12 patients (Figure 3). A closed-funnel configuration was observed in nine eyes of seven patients, an open-funnel configuration in one eye of one patient, diffuse high echogenicity in nine eyes of seven patients, PFV-like findings in two eyes of two patients, a pseudoglioma-like appearance in one eye of one patient, and a falciform retinal fold in one eye of one patient.

**Figure 4:**
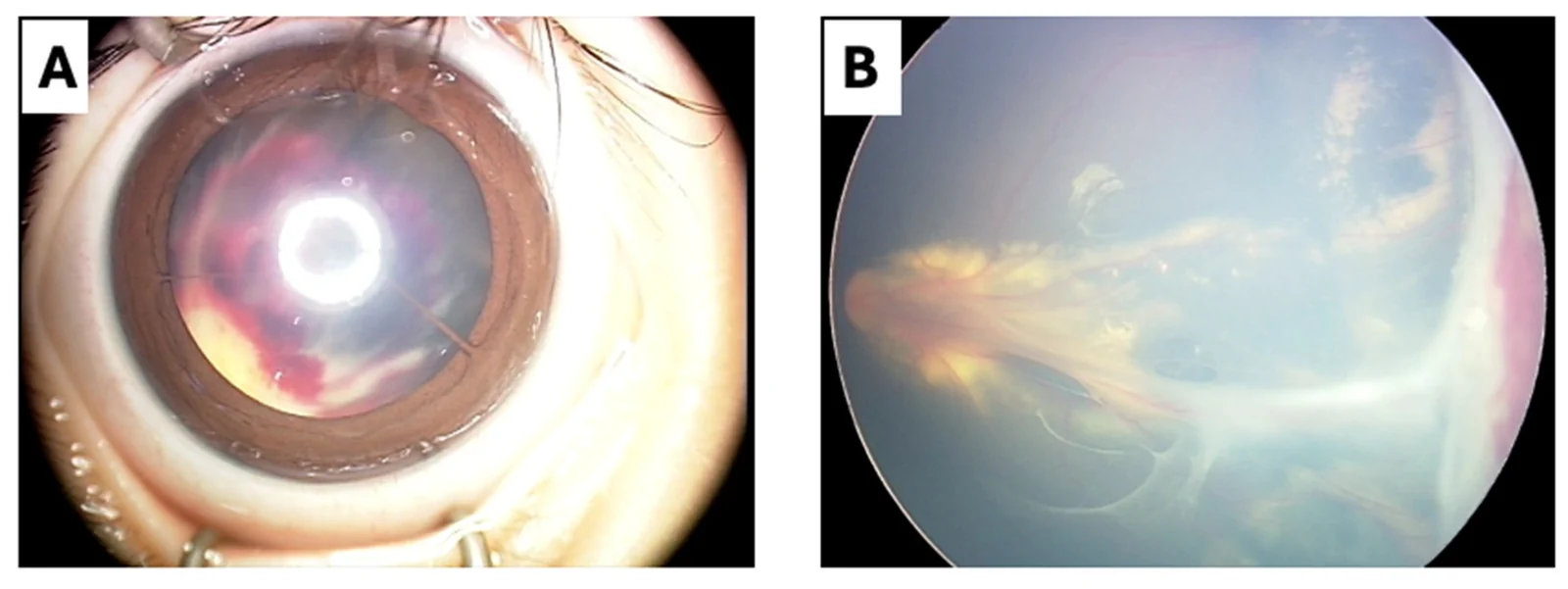
Atypical Norrie disease with relatively late presentation and asymmetric retinal detachment. Patient 7 first presented at 3 years of age with stage 5B (based on reference [18]) in the right eye (**A**) and stage 4 disease in the left eye (**B**).

Regarding interocular differences in disease severity, four patients from four families had stage 5B disease in one eye and stage 4 disease in the fellow eye. One of these patients first presented at 3 years of age, representing a relatively late presentation with stage 5B/stage 4 disease (Figure 4) [16]. In another patient, one eye had stage 5C disease, and the fellow eye had stage 2 disease at the initial examination. The stage 2 eye progressed to stage 4 five months after laser photocoagulation of the avascular retina and subsequently underwent vitrectomy [14,19].

Treatment was performed in 21 of the 28 eyes from 13 patients (75%). Retinal laser photocoagulation was performed in four eyes of four patients (14%), and surgical treatment was performed in 18 eyes of 11 patients, including one laser-treated eye (64%). Among the 13 eyes that underwent vitrectomy, 12 underwent vitrectomy combined with lensectomy, whereas one underwent lens-sparing vitrectomy. Lensectomy alone was performed in five eyes of three patients to improve or maintain corneal transparency. Among patients followed for at least 1 year, the final visual acuity was no light perception in all eyes, except for two laser-treated eyes that still had light perception or better visual acuity.

### 3.2. Cases in Japan

**Figure 5:**
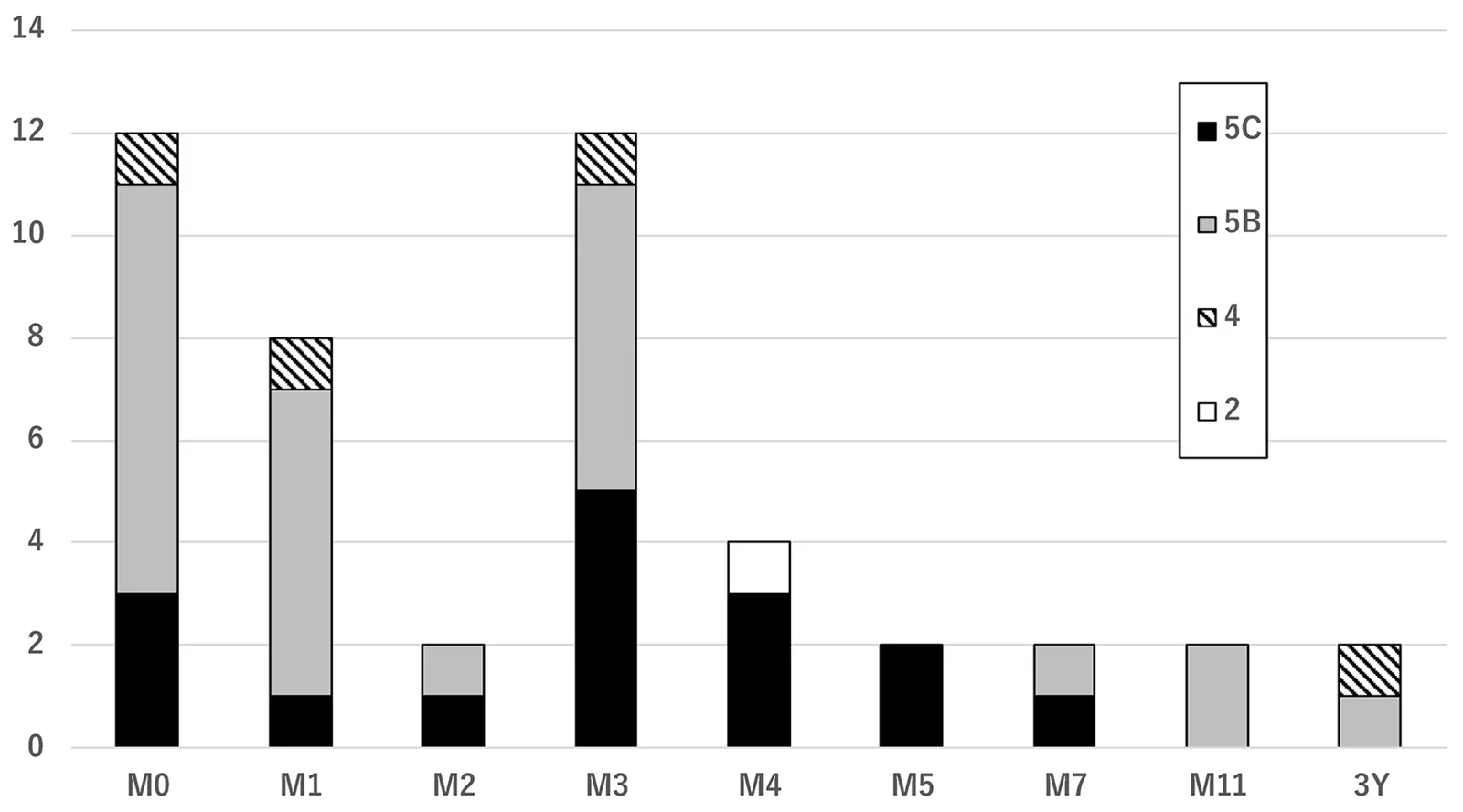
**Age at the initial ophthalmic examination and disease stage in 23 Japanese patients with Norrie disease.** Data include the present cohort and all previously reported Japanese cases identified through a systematic literature review. Most eyes had stage 5 retinal detachment by 2 months of age. M and Y indicate months and years of age, respectively. The y-axis represents the number of eyes. Stage 5 is subclassified based on reference [18].

**Figure 6:**
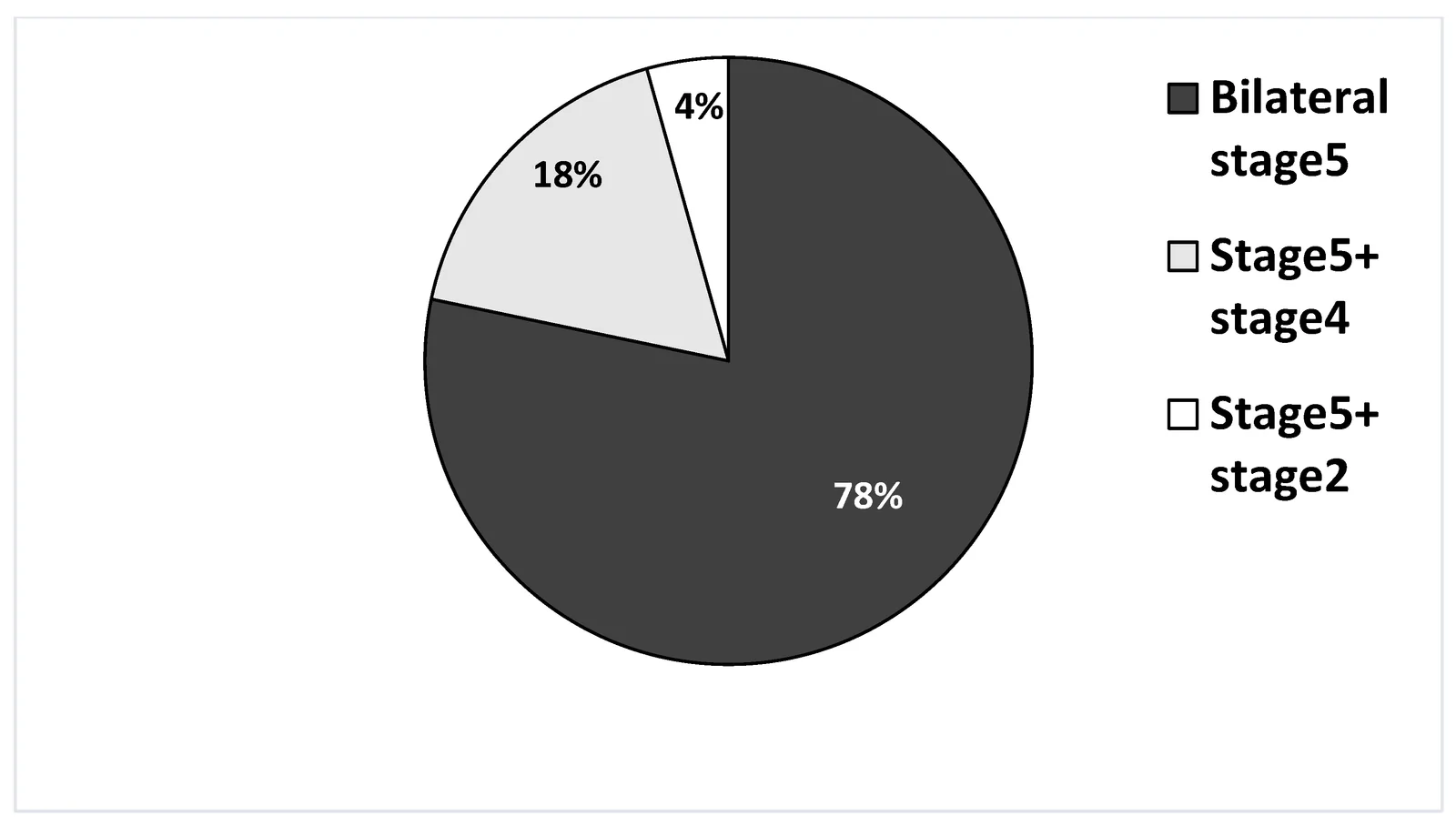
**Interocular asymmetry in 23 Japanese patients with Norrie disease.**

The literature search identified an additional 13 patients from six families. Combined with the present cohort, a total of 29 patients from 19 Japanese families were identified (Figure 2). One patient without *NDP* genetic testing was excluded. After excluding six patients from six families because of lack of genetic testing, insufficient clinical information, or presentation in adulthood, 23 patients from 16 families were included in the age-based analysis (Table 2 and Table 3). Among the six patients diagnosed before 1 month of age, 11 of 12 eyes were classified as stage 5 and one eye as stage 4. Among the four patients diagnosed at 1 month of age, seven of eight eyes were classified as stage 5 and one eye as stage 4 (Figure 5). With respect to interocular asymmetry, 18 patients, accounting for 78% of the analyzed cohort, had bilateral stage 5 disease at diagnosis. Four patients had stage 5B disease in one eye and stage 4 disease in the fellow eye, whereas one patient had stage 5C disease in one eye and stage 2 disease in the fellow eye. Thus, marked interocular asymmetry was absent in most patients (Figure 6).

## 4. Discussion

In this study, we summarized the acute clinical manifestations of Japanese patients with Norrie disease at their initial ophthalmic examination. Because Norrie disease is an *NDP*-associated vitreoretinopathy closely related to FEVR, we based our staging system on the FEVR classification. However, given that most patients presented with advanced disease, the original A/B subclassification for the milder stages was omitted, whereas stage 5 was expanded by adding stage 5C based on ICROP3 to distinguish end-stage disease. With the exception of one eye in one patient, all eyes were classified as FEVR stage 4 or higher, indicating retinal detachment involving the macula at presentation. Long-term visual outcomes were poor regardless of treatment. At the most recent follow-up, all but two eyes had progressed to no light perception. The two exceptions were stage 4 eyes treated with laser photocoagulation, both of which retained vision better than light perception at 1 year of follow-up. Although anatomical improvement, including retinal reattachment or maintenance of an open-funnel configuration, was achieved temporarily in some cases, long-term preservation of visual function remained difficult. Corneal opacity appears to develop as intraocular proliferative tissue compresses the crystalline lens anteriorly, resulting in shallowing and eventual obliteration of the anterior chamber, followed by iridocorneal adhesion. In contrast, eyes with a well-maintained anterior chamber may represent cases in which the anterior displacement of the lens caused by retrolental proliferative tissue has been relieved. On B-scan ultrasonography, eyes corresponding to stage 5B or 5C retinopathy of prematurity typically exhibited a closed-funnel retinal configuration. However, when extensive subretinal hemorrhage produced diffuse high echogenicity, delineation of the retinal configuration became difficult, limiting accurate assessment by ultrasonography.

Walsh et al. suggested that very early vitrectomy may preserve light perception in patients with Norrie disease, based on the observation that light perception is often lost by 3 months of age [6]. In this study, with the possibility of very early surgical intervention in mind, we investigated how soon after birth Norrie disease must be diagnosed to detect relatively early-stage retinal abnormalities. At 0 or 1 month of age, however, all eyes had already progressed to stage 5, except for two eyes from two patients that were classified as stage 4.

By combining our cohort with all previously reported Japanese cases, we demonstrated that most patients already had bilateral stage 5 retinal detachment within the first 2 months of life. These findings indicate that the therapeutic window for effective intervention, including laser photocoagulation and vitreoretinal surgery, is extremely narrow, as most patients already have advanced retinal disease at the time of diagnosis. In our cohort, the decision whether to proceed with surgery was ultimately left to the parents after discussion of the expected benefits and risks. The families of patients with stage 5C disease happened to elect surgical treatment more frequently than those of patients with stage 5B disease.

The spectrum of *NDP* variants identified in Japanese patients indicates that each family harbored a distinct variant [9,11,12,13,14,15,16]. Case 12 (the novel case) had an exceptionally rare combination of two X-linked recessive disorders, Norrie disease (Xp11.3) and hemophilia A (Xq28). Although the coexistence of two X-linked recessive disorders may raise the possibility of an underlying chromosomal rearrangement, this appears unlikely because the identified *NDP* variant was a point mutation. Excluding two variants that have not yet been published, 14 different variants have been reported, of which seven were predicted loss-of-function (null) variants, including nonsense, frameshift, splice-site, initiation codon, or exon deletion variants (p.Met1?, p.His4Argfs, p.Tyr44Ter, p.Phe30Phefs, p.Gly112Cysfs, c.175-1G>A, and deletion of exon 2). The remaining seven variants consisted of missense or in-frame deletion variants, including four predicted to result in the gain or loss of a cysteine residue (p.Cys95Arg, p.Arg38Cys, p.Cys65Tyr, and p.Cys126Gly) and three other variants (p.Ile18Lys, p.Arg97Pro, and p.Gln99_Thr100del). One patient carrying p.Ile18Lys also harbored a *TSPAN12* variant, representing digenic inheritance [16]. Among Japanese patients with Norrie disease, missense variants not involving cysteine residues and in-frame deletion variants accounted for only approximately 21% of all variants. Consistent with the findings of Wawrzynski et al. [20], such variants were relatively uncommon, and most cases were attributable to variants predicted to cause severe loss of norrin function.

Although most patients diagnosed with Norrie disease in the present study exhibited the typical severe phenotype, some showed marked interocular asymmetry or were diagnosed later in life. In such cases, differentiation from XL-FEVR is necessary. Characterization of the underlying *NDP* genotype may provide useful information for distinguishing between these two phenotypes. Wawrzynski et al. [20] reviewed approximately 200 previously reported *NDP* variants and found that, although disease severity varied among variants, each individual variant was generally associated with a relatively consistent phenotype. Nevertheless, Norrie disease and XL-FEVR have also been proposed to represent a phenotypic continuum. Further studies are therefore needed to determine whether the two conditions can be reliably distinguished on the basis of clinical findings alone. Bilateral congenital retinal non-attachment and bilateral persistent fetal vasculature caused by other etiologies should also be considered in the differential diagnosis [21,22].

Laser photocoagulation has been performed in selected patients with relatively mild or atypical manifestations, such as falciform retinal folds or stage 2 disease [14,19]. However, progression may still occur despite treatment. In one eye that underwent laser photocoagulation for stage 2 disease, retinal detachment progressed to stage 4 within 5 months after panretinal photocoagulation, suggesting that even eyes with apparently mild disease at diagnosis require careful long-term follow-up [19]. In patients with advanced disease, lensectomy may be performed to manage corneal opacity, whereas vitreoretinal surgery may be attempted to preserve visual function. Nevertheless, the long-term visual prognosis remains extremely poor. Chow et al. [23] reported a favorable outcome in a patient who underwent prenatal genetic diagnosis followed by immediate postnatal laser photocoagulation. These findings suggest that preservation of visual function in patients with Norrie disease may depend on diagnosis before the onset of advanced retinal pathology. Future studies should therefore evaluate the role of prenatal genetic diagnosis, together with very early postnatal screening and prophylactic therapeutic intervention, in improving visual outcomes.

## 5. Conclusions

We characterized the overall clinical features of Japanese patients with Norrie disease by analyzing our own cohort together with all previously reported cases in Japan. At the time of initial diagnosis, most patients already had advanced disease, with severe retinal detachment that was difficult to treat. Although laser photocoagulation may be effective in a limited number of selected patients, opportunities for therapeutic intervention are extremely limited once the disease has progressed. Increasing awareness of Norrie disease among not only pediatric retina specialists but also general ophthalmologists may facilitate earlier recognition and diagnosis. Because some patients exhibit marked interocular asymmetry, careful differential diagnosis from XL-FEVR and PFV remains essential.

## Figures and Tables

**Table 1:** Classification of Norrie disease based on a modified familial exudative vitreoretinopathy staging system with stage 5 subdivision according to the International Classification of Retinopathy of Prematurity, Third Edition.

Stage *	Description
1	Peripheral avascular retina
2	Peripheral avascular retina with retinal neovascularization
3	Partial retinal detachment sparing the macula
4	Partial retinal detachment involving the macula or congenital absence of macular formation without total retinal detachment
5 **	Total retinal detachment
5A	The optic disc is visible on ophthalmoscopy
5B	The optic disc is obscured by retrolental fibrovascular tissue or closed-funnel retinal detachment
5C	End-stage disease characterized by secondary glaucoma with anterior lens displacement and/or corneal opacity

* Simplified from the FEVR staging system [17] by omitting the original A/B subclassification. ** The original FEVR stage 5A/5B subclassification (open- and closed-funnel total retinal detachment) was replaced with the International Classification of Retinopathy of Prematurity, Third Edition (ICROP3) classification [18].

**Table 2:** Early clinical manifestations and visual prognosis in Norrie disease.

Family and Patient ID	Age at Initial Examination	R/L	Corneal Transparency	Anterior Chamber Depth	Stage at Initial Examination *	Ultrasonography Findings	Surgery	Final Visual Acuity	Comment [Reference]
1	M3	R	opacity	none	5C	closed funnel	V	NLP	[16]
		L	clear	shallow	5B	DHE	V	NLP	
2	M1	R	clear	shallow	5B	closed funnel	Conservative management	NLP	[16]
		L	clear	shallow	5B	closed funnel	V	NLP	
3	M3	R	opacity	normal	5C	DHE	LS	NLP	[16]
		L	clear	shallow	5B	closed funnel	V	NLP	
4-1	M2	R	clear	shallow	5B	closed funnel	LS	NLP	[14,15]
		L	opacity	none	5C	closed funnel	LS	NLP	
4-2	M0	R	clear	normal	4	PFV-like	V	NLP	[14]
		L	clear	normal	5B	closed funnel	V	NLP	
5	M0	R	clear	normal	5B	open funnel	Conservative management	NLP	[16]
		L	edema	normal	5C	DHE	V	NLP	
6	M4	R	opacity	none	5C	DHE	LS	NLP	[14,15]
		L	opacity	none	5C	DHE	LS	NLP	
7	3Y	R	clear	normal	5B	NA	LSV	NLP	Affected carrier mother, [16]
		L	clear	normal	4	NA	V	NLP	
8	M1	R	opacity	none	5C	NA	LS→V	NLP	[16]
		L	clear	shallow	5B	NA	V	NLP	
9	M4	R	opacity	shallow	5C	Pseudoglioma-like	Conservative management	NLP	[14,19]
		L	clear	shallow	2 **	NA	PC→V	NLP	
10-1	M11	R	clear	shallow	5B	closed funnel	Conservative management	NLP	Affected carrier mother, [Submitted manuscript]
		L	clear	normal	5B	DHE	PC	NLP	
10-2	M1	R	clear	shallow	5B	closed funnel	Conservative management	NLP	Affected carrier mother, [Submitted manuscript]
		L	clear	shallow	4	falciform fold	PC	LP	
11	M3	R	clear	shallow	4	PFV-like	PC	LP	[Submitted manuscript]
		L	clear	shallow	5B	DHE	V	NLP	
12	M1	R	clear	shallow	5B	DHE	Conservative management	NLP	[This study]
		L	clear	shallow	5B	DHE	Conservative management	NLP	

DHE, diffuse high echogenicity; L, left eye; LP, light perception; LS, lensectomy; LSV, lens-sparing vitrectomy; M, months; NA, not available; NLP, no light perception; PC, photocoagulation; PFV, persistent fetal vasculature; R, right eye; V, vitrectomy; Y, years. * Stage 5 is subclassified based on the International Classification of ROP, Third Edition [18]. ** Retinal detachment progressed from stage 2 to stage 4 at 9 months of age.

**Table 3:** Early clinical manifestations and visual prognosis in previously reported Norrie disease (literature review).

Family and Patient ID	Age at Initial Examination	R/L	Corneal Transparency	Anterior Chamber Depth	Stage at Initial Examination *	Ultrasonography Findings	Surgery	Final Visual Acuity	[Reference]
13-1	M3	R	Clear	Shallow	5B	NA		NLP	[7,8,9]
		L	Clear	Shallow	5B	NA		NLP	
13-2	M0	R	Clear	Normal	5B	NA		NLP	[9]
		L	Clear	Normal	5B	NA		NLP	
14-1	M3	R	Opacity	Shallow	5C	NA		NLP	[8,9]
		L	Opacity	Shallow	5C	NA		NLP	
14-2	M3	R	Clear	Normal	5B	NA		NLP	[9]
		L	Opacity	Shallow	5C	NA		NLP	
15-1	M0	R	Opacity	NA	5C	Funnel-shaped retrolental mass	V	NLP	[12]
		L	Opacity	NA	5C	Funnel-shaped retrolental mass		NLP	
15-2	M0	R	Clear	NA	5B	Funnel-shaped retrolental mass		NLP	[12]
		L	Clear	NA	5B	Funnel-shaped retrolental mass		NLP	
16-1	M7	R	Clear	Shallow	5B	Total RD		NLP	[13]
		L	Opacity	Shallow	5C	Total RD		NLP	
16-2	M5	R	Opacity	Shallow	5C	Total RD		NLP	[13]
		L	Opacity	Shallow	5C	Total RD		NLP	
16-3	M0	R	Clear	Shallow	5B	Total RD		NLP	[13]
		L	Clear	Shallow	5B	Total RD		NLP	

L, left eye; LP, light perception; M, months; NA, not available; NLP, no light perception; R, right eye; RB, retinoblastoma; RD, retinal detachment; V, vitrectomy. * Stage 5 is subclassified based on the International Classification of ROP, Third Edition [18].

## Data Availability

The data that support the findings of this study are available from the corresponding author upon reasonable request, subject to restrictions related to the protection of personal information.

## Abbreviations

FEVR	familial exudative vitreoretinopathy
NDP	Norrie disease protein
XL-FEVR	X-linked familial exudative vitreoretinopathy
PFV	persistent fetal vasculature
ROP	retinopathy of prematurity
ICROP3	International Classification of Retinopathy of Prematurity, Third Edition
